# Using an Empathetic Approach to Explore Technology Readiness and Needs for Digital Services to Assist People with Dementia

**DOI:** 10.3390/ijerph21081023

**Published:** 2024-08-02

**Authors:** Mille Aagaard Engblad, Emilie Pind Herstal, Emilie Kauffeldt Wegener, Lars Kayser

**Affiliations:** Department of Public Health, Faculty of Health and Medical Sciences, University of Copenhagen, Øster Farimagsgade 5, 1353 Copenhagen K, Denmarkemilie.wegener@sund.ku.dk (E.K.W.);

**Keywords:** dementia, empathetic approach, health technology readiness, innovative approach, collaboration, inclusion

## Abstract

This qualitative study investigates technology readiness, i.e., self-management, social support, and digital health literacy, in people with dementia (PwD). PwD are difficult to recruit; therefore, we used an empathic approach to recruit and conduct interviews. The interviews with seven participants with dementia and two informal caregivers, guided by the READHY framework, reveal nuanced insights into their experiences. Participants demonstrate varying degrees of self-management, with informal caregivers playing pivotal roles in facilitating activities and supporting overall well-being. Cognitive challenges, such as concentration and communication difficulties, are prevalent, highlighting the importance of robust support systems. Internal and external support networks significantly influence social integration, yet societal misconceptions impede inclusion, exacerbating feelings of isolation for both participants and caregivers. Limited interaction with technology is observed, primarily reliant on caregivers for assistance. Technology may hold potential for enhancing independence and alleviating caregiver burden. As an empathetic approach eased recruitment and communication with PwD, we recommend using this approach for future studies to include participants who otherwise would not be recruited. Given that the number of participants in this study is limited to only seven PwD with moderate to severe cognitive impairment, further investigation using mixed methods, including the READHY framework, and a larger number of participants is needed to examine the generalizability of the findings.

## 1. Introduction

The population aged 60 and over is expected to double to 2.1 billion by 2050 [1]; similarly, the number of people aged 80 and older is expected to triple to 462 million people between 2020 and 2050 [1]. Due to this increased life expectancy, dementia has become a growing concern, and it is expected that up to 131.5 million people globally will be living with dementia by 2050 impacting millions of people and their families [2].

Dementia is a neurological disorder and is the term for the conditions where cognitive functions are impaired [3]. People with dementia (PwD) experience problems with memory, reasoning, impaired initiative, communication, and sense of place [4]. These problems can become so profound that they can affect the ability to cope with everyday life, as basic daily activities become a challenge [2].

Dementia can also cause behavioral disturbances, where personality changes can occur and interactions with other people will change. This may lead to social disability, which can be characterized by disadvantages and exclusion that PwD face due to impaired social perceptions and structural barriers related to their condition, often resulting in marginalization of PwD and their informal caregivers [5].

Addressing this risk of marginalization and social exclusion among PwD requires practical interventions aimed at improving the quality of life for both PwD and their informal caregivers. Recognizing the specific forms of exclusion and disability associated with dementia is crucial for fostering a more inclusive environment. An understanding of determinants for and mechanisms causing stigmatization, exclusion, and disability when living with dementia is needed to be able to provide active support [5].

New digital services have opened opportunities to not only ease the burden on informal caregivers but also support PwD to live more independently at home [6]. Implementing technological devices can support daily function, navigation, and mobility in everyday life [6]. A study by Lorenz et al. [7] shows that most of the digital technologies developed for PwD aim to facilitate safety and security for both PwD and their informal caregivers. These technologies are not yet widespread as a practical application in daily life due to various factors, such as lacking awareness of the options and utility, frequent changes in needs and requirements to the solutions due to fast cognitive decline, and limited documentation of cost efficiency. This often leads to reliance on everyday products, such as re-purposed mobile phones, proved more prevalent to meet the needs of PwD because they are more accessible and familiar to the users. Also, GPS systems may help PwD to navigate safely and alert caregivers if they wander, while voice prompts from smart speakers in the home can assist in daily activities like medication reminders. Panic buttons provide quick emergency assistance, and motion sensors detect falls and unusual movements, ensuring safety [7]. 

Properly designed assistive technologies can help enhance independence for PwD and support informal caregivers in delivering comprehensive care [8]. However, thus far, designing solutions for this group remains highly complex, with inadequate methods for eliciting technology preferences from individuals with cognitive impairments [9]. Currently, there exists a lack of knowledge regarding recruitment and involvement of PwD and their informal caregivers related to the design of new technologies and digital services that suits their special needs [10]. In response to these challenges, innovative thinking is needed, and new ways of approaching the question of whether technology can play a useful role in the care and support of PwD and their informal caregivers is required. 

In the EU-funded project ‘Smart Inclusive Living Environments’ (SMILE) [11], an aim is to identify new ways to include and give voice to PwD to inform the design of new digital services and technologies, based on their needs and preferences. In response to this, the aim of the study reported here is to find ways to best approach PwD and investigate their technology readiness and needs for support and assistance. The multidimensional Readiness and Enablement Index for Health Technology (READHY) framework was used to guide the development of a semi-structured interview guide and the qualitative analysis of the interviews to assess PwD’s readiness for adopting technology [12]. 

## 2. Materials and Methods

### 2.1. Population and Setting

The study population consists of PwD with moderate to severe cognitive impairment who were willing to participate in semi-structured interviews and had varying levels of technology familiarity. Exclusion criteria comprised those unable to provide informed consent, severe cognitive impairment hindering communication, or unwillingness to discuss health and technology experiences. The sample group comprised seven participants and two informal caregivers (Table 1). 

The setting of the semi-structured interviews varied across different geographical locations, two took place in the homes of the participants, and five took place in nursing homes where the participants live. The interviews were planned with scheduling flexibility to accommodate the participants and informal caregivers, to ensure inclusivity and gather diverse perspectives efficiently.

The first interview featured participant 1 and their informal caregiver. The second interview featured the informal caregiver of participant 2; due to participants 2’s condition and the anticipated adverse reaction to the interview process, the interview was conducted solely with the informal caregiver. The subsequent five interviews featured participants residing in nursing homes. Participants 1 and 2 are actively engaged in support networks including the Alzheimer’s Association and had regular contact with a dementia coordinator. In contrast, the remaining five participants, who are residents of nursing homes, primarily interacted with the formal caregivers at the nursing homes.

### 2.2. Recruitment

Initially, the focus was on recruiting people diagnosed with dementia living at home with an informal caregiver, but this focus in the recruitment process faced several challenges. For example, it was difficult to find PwD in general and even more challenging to find couples still living together where one had dementia. Additionally, it was difficult to find couples willing to participate. In general, getting access to communities of PwD and informal caregivers was problematic. When contacting nursing staff or dementia coordinators, we often received negative responses. Common feedback included concerns that participation would be too burdensome for PwD and their informal caregivers, or that the decision was beyond the respondents’ authority and required approval from higher-level management. These recruitment challenges led to an expansion of the target population description to include all PwD regardless of housing situation, care setting, and their informal caregivers. To access potential participants, gatekeepers (Dementia Coordinators, nursing staff, and community event planners) were identified to facilitate the project goals [13]. Incorporating established connections proved effective, sparking interest from multiple participants in the process.

While we experienced limited success reaching out through social media platforms, contacting staff directly at nursing homes presented challenges due to the sensitivity of potentially disrupting the daily routines of PwD. Overcoming this, a colleague’s referral connected us with a couple navigating dementia challenges. Personal contacts facilitated connections with nursing homes, where preliminary discussions ensured participant understanding and comfort, laying the foundation for trust in the interview process. Collaborating with dementia coordinators led us to engage with an activity center and an evening school in Copenhagen. During an event at the school, we introduced our project. This resulted in an informal caregiver showing an interest in participating, which led to an expansion of our participant network.

The formal caregivers at the nursing homes had a pivotal role in connecting us with the five participants living in nursing homes. Their guidance ensured that only people with dementia conditions, who were unlikely to trigger unpleasant reactions during interviews, were selected. This strategic approach not only safeguarded the well-being of the participants but also provided a nuanced perspective on technology readiness within the context of diverse support structures.

### 2.3. Data Collection

In two preliminary evaluations in Denmark and the Netherlands, respectively, the quantitative READHY instrument [2] was applied during interviews with PwD. Here, we found that it was not a feasible way to address and categorize participants with moderate to severe cognitive impairment. Therefore, we have used READHY as a framework to inspire and inform the interview guide and analysis for the semi-structured interviews (Table S1).

### 2.4. Semi-Structured Interviews

The purpose of the semi-structured interviews is to identify and map the participant’s attitudes and experiences with health, support, networks, and technology, which contributed to a needs assessment for person-centered support systems, building on the READHY instrument [14]. READHY is designed to unveil the readiness of individuals towards digital solutions and serves as a guiding principle to probe into users’ preparedness for utilizing digital tools [15]. READHY encompasses three main domains, namely (1) Self-management: This domain evaluates individuals’ capacity and resources to manage their health independently. (2) Social support: It encompasses the perceived support from networks, including both familial and healthcare professional support. (3) Digital health literacy: This domain focuses on individuals’ motivation and ability to use and trust health-related technologies [15]. 

The interview guide, shaped by READHY, was designed with the specific needs of PwD in mind, to be flexible, ensuring a comprehensive exploration of participants’ perspectives. This approach prioritized natural and informal interviews, supporting a personal and empathetic connection, essential in understanding health, support networks, and technology use among PwD. 

### 2.5. Empathic Approach 

In this study, an empathic approach was used, inspired by Wright and McCarthy’s methodology [16]. This approach was important in establishing a profound connection with the participants, transcending the conventional role of researchers as mere gatherers of information. Our emphasis was on recognizing the participants as individuals with unique perspectives, personal positions, and rich emotional experiences.

Throughout the semi-structured interviews, our commitment to an empathic approach became evident in our active presence and engagement. We conscientiously cultivated a welcoming and secure environment, choosing to conduct interviews in the intimate setting of the participants’ homes, responding to their expressed need for familiarity and comfort [16]. This decision not only facilitated open communication but also allowed us to observe and understand aspects of their daily lives that may not have been apparent in a more formal setting.

Active listening was a cornerstone of our approach, as we strived to truly comprehend the nuances of our participants’ thoughts and perspectives. We avoided leading questions, ensuring that the dialogue remained organic and reflective of the participants’ genuine experiences. Employing clear and concise language, we gave ample time for responses and adapted our questions to accommodate the needs of each participant.

### 2.6. Data Analysis and Codebook 

Content analysis was conducted using an abductive approach to analyze the qualitative data extracted from the semi-structured interviews [17]. This methodological choice was driven by the intention to leverage the READHY framework to delve into and elucidate the research questions [18]. The foundation of data analysis lies in the creation of a codebook, essential for organizing and categorizing the collected data. Nvivo (version 14; Lumivero), a qualitative analysis tool, was employed for coding purposes.

The READHY framework served as the scaffolding for the codebook development. With its 13 dimensions across three main domains, READHY provided a comprehensive lens to explore potential barriers and resources of individuals [15]. Drawing inspiration from these dimensions and experience from previous study, the codebook was designed to delve deeper into the data and gain a nuanced understanding of PwD. In the following, we describe the reasoning behind the selected subcategories and the derived codes (Table 2). To explore the first main category self-management, insights from heiQ4 (constructive attitudes and approaches) and heiQ8 (emotional distress) were integrated and resulted in the subcategory, impact of condition. These dimensions shed light on the emotional impact, coping strategies, and overall well-being concerning the condition [19]. Additionally, dimensions like heiQ3 (self-monitoring and insight) and heiQ5 (skills and technique acquisition) were utilized to understand self-management and its implications on health-related behaviors [19] which resulted in the subcategory capabilities and resources. For the second main category, social support, the influence of social life and networks on technology readiness were explored; two dimensions, HLQ1 (feeling understood and supported by healthcare providers) and HLQ4 (social support for health), were incorporated, and resulted in the subcategories external support and internal support, respectively. These subcategories capture the support from healthcare providers and social networks, crucial for avoiding social disability in relation to use of technology and everyday activities [2]. For the third main category, digital health literacy, a focus on the role of technology necessitated an examination of individuals’ attitudes, motivations, and competencies toward digital services. Insights into individuals’ access to digital services were obtained through the inclusion of eHLQ3 (ability to actively engage with digital services), eHLQ6 (access to digital services that work), and eHLQ7 (digital services that suit individual needs), which resulted in the subcategory competencies and insights related to engagement from eHLQ5 (motivated to engage with digital services), which resulted in the subcategory motivation to engage in digital services [2].

The dimensions eHLQ1 (using technology to process health information), eHLQ2 (understanding of health concepts and language), and eHLQ4 (feel safe and in control) regarding user’s ability to find information, ability to understand health concepts and language, and feeling safe and in control of how data are used, were not used in the coding. We did not find the dimensions eHLQ1 and eHLQ2 to be relevant, since we did not focus on what PwD use technology for, but rather the broader understanding of how they use the technology. The dimension eHLQ4 was not included since there was not a focus on data security. 

During the coding process, a new subcategory emerged under the main category of social support: community support along with a code. Additionally, a new code was identified under digital health literacy, in the subcategory competencies: limitations or challenges related to technology. These are highlighted in grey (Table 2).

### 2.7. Ethical Considerations and Informed Consent

Informed consent was obtained from all humans participating in this study. Our research has followed the declaration of Helsinki’s ethical principles for medical research involving human subjects. In Denmark, health science questionnaire surveys and interview studies that do not involve human biological material (section 14) of the Danish Act on Committees do not require reporting or approval from the Danish National Centre for Ethics [20].

## 3. Results

In the following section, we present our results structured according to the READHY framework, which informs this study in the categories of self-management, social support, and digital health literacy. The data are based on interviews conducted with seven individuals diagnosed with moderate to severe cognitive impairment and two informal caregivers.

### 3.1. Self-Management

#### 3.1.1. Impact of Condition

The participants are in their everyday lives affected to varying degrees by their dementia condition. Situations quickly arise where they feel frustrated or overwhelmed, especially in stressful situations where there is noise or many impressions.

*P1: “Yes, but when four people are talking at the same time, it’s a problem. Because you want to answer them all, but you can’t”.*

*EM: “Do you get frustrated with the sound?”*

*P1: “Yes, I don’t feel I can follow what’s going on, that is fucking annoying”.*

*EM: “How do you feel when there is a lot of noise?”*

*P1: “I usually go”.*

Such situations can make it challenging to be in social contexts, as well as places where there are demands for a specific behavior and where certain norms and rules must be followed. These requirements can be difficult to follow for PwD, which can create unpleasant situations for both PwD and informal caregivers, which makes it difficult to navigate in society.

It was difficult to get an insight into the impact of the condition of participants three to seven at the nursing home, as several expressed that they did not feel that they had any challenges.

*EM: “So how do you think your general health is?”*

*P4: “Yes, I am very satisfied”.*

*P5: “No, not at all. Well, I have no health problems. I have not. Both health and mood. I have no problems with that”.*

It was also expressed several times by P1 that he did not feel either affected or worried about his condition. This insight was counterargued by the informal caregiver.

*EM: “So it doesn’t matter much to you?”*

*P1: “No, I kind of don’t care”.*

*IC1: “No, you like that when you get lost, you can get in touch with me”*

*P1: “Yes, that’s right”.*

Several times in the first interview, P1 repeats or confirms what IC1 says. The interviews therefore give the impression that there is a tendency for PwD to repeat or confirm what has previously been said.

#### 3.1.2. Capabilities and Resources

Several of the participants had challenges with cognitive skills, mainly concentration, coordination, and communication.

*P1: “Well, I can’t coordinate them anymore, the numbers. They are not quite as good as they used to be”.*

*IC2: “I would say that he has difficulty finding the words. He couldn’t explain what he meant. It was something with one foot, the carpet, or something? He finds it difficult to express himself, sometimes”.*

We saw a tendency for the participants without informal caregivers to handle the deteriorating cognitive skills by either avoiding situations or tasks where they would be challenged or by having an optimistic attitude towards the things they were previously capable of.

*P6: “What am I listening to? Radio, television. Then I see what is happening today. But otherwise, I don’t see much. I don’t sit and watch movies and such. I can’t do that anymore. That is also okay”.*

Through interactions with the informal caregivers, the participants can find solutions which allow them to continue activities that are otherwise challenged by the changes in the participants’ cognitive skills.

*EM: “So that’s also why you play Yatzy with animals on it, it’s easier than having numbers?”*

*P1: “Yes, pictures, are better”.*

*IC1: “All those numbers, ugh, it’s difficult. But when it’s animal yatzy… But we have fun with it anyway”.*

It proposes challenges that the dementia condition gradually develops, as this can cause a change in needs. P1′s ability to navigate has deteriorated within the last year, which is why they are now dependent on taxis.

*EM: “What did you do last year?”*

*IC1: “He could cycle there himself, he could cycle well there”.*

*P1: “I could take the bike there”.*

*EM: “So it’s within the last year [P1 can’t ride a bike]?”*

*IC1: “Yes, the last year and a half”.*

*P1: “Yes oh, yes yes. I’m not that good at finding my way anymore”.*

It was also observed that some participants’ dementia diagnosis worsened in a matter of months.

*EM: “So there has been a change in his condition here in the last few months?”*

*IC2: “Yes, there is that too. As P1 also said here the last time, we were together, P1 could feel the difference between Christmas and now”.*

We saw a tendency to express a need for activity and movement. Several participants identified activity as something positive, and that it was important for them to get outside.

*EM: “Would you rather be at home or the activity home?”*

*P1: “I’d rather be in the activity home, where I do activities, it does a lot. I love going for walks”.*

### 3.2. Social Support 

The analysis of the coding revealed that the codes related to the subcategories of internal support and external support were often intertwined in the results. This overlap made it challenging to separate the two categories. Therefore, we have combined them and present them together under the term role of individual support.

#### 3.2.1. Role of Individual Support

Internal support has a significant influence on PwD’s well-being in everyday life. The participants at the nursing home often mentioned their informal caregivers, children, and grandchildren, in connection with their daily contact, and highlighted this contact as a positive aspect in their life and for their well-being.

*P3: “He calls every morning and wishes me a good day. Good son I have, and daughter-in-law. We have always been friends, and she comes with us when we travel”.*

*P6: “My daughter, she lives here somewhere. Her children have left home. They live in Copenhagen. They come to visit me. They are nice to that”.*

The participants still living in their own homes have a greater dependence on practical and social support from informal caregivers, mainly from their cohabiting partner.

*IC2: “I also stand and direct him and help him remember various things when he stands there. Then he stands there and puts water on his body and I say that he must also remember to wash his body, remember to put soap on, wash his hair, and rinse his hair. He forgets that he has to do one thing and the other”.*

The informal caregivers describe that supporting the participants is resource-intensive and places high demands on their income and ability to manage them. In this context, the informal caregivers expressed a need for additional support and help from an external network. Support from an external network can provide relief for informal caregivers, as it helps relieve the burden of caregiving. However, mistrust towards external support services can create frustration and powerlessness. This powerlessness can lead to a fear of asking for help.

*IC1: “He asked about everything and then he [the doctor] got mad at me because I answered. I said, well, he can’t remember it himself. Well, if I couldn’t resist answering, I could go out. Then I thought, what the hell is he thinking? Are you sure it won’t get better? [asked the doctor], then I thought no, I can’t do this. No, but you’re a doctor and you ask if Alzheimer’s is getting better, right? Then you give up a little, right?”*

*IC1: “But to think that you have to sit and be afraid to call for something like that. Isn’t that terrible? I feel like a real chicken, it’s not because I’m afraid to call, I’m afraid of the answer”.*

The informal caregivers describe that their support does not only entail practical tasks but also facilitation of social activities for the PwD.

*IC1: “He would not voluntarily be social today, he would very much like to sit in here and watch television all the time, if it was possible. And he could do that too, then I could sit here and wait, then it wouldn’t be many years before he could do nothing. So, I have to force him or push him into situations”.*

There was a general tendency in the interviews for informal caregivers to become the spokesperson for PwD, as it is natural for them to compensate when the PwD have difficulties expressing themselves. It can therefore be difficult to separate the PwD’s insights and experiences from the informal caregiver’s perspective.

*EM: “But P1, in your everyday life. What do you think is best and what do you think is most difficult? Is there something that annoys you?”*

*P1: “I don’t know right now… I can’t figure it out”.*

*IC1: “Can I help you?”*

*P1: “Yes, you may”.*

*IC1: “What annoys you, I know that, and that’s why I don’t do it when you’re home. It’s all that crafty stuff that you’ve always been good at. If someone comes and helps me/us with it when he’s at home, he can’t! Because ‘I can do it myself, let it be’ [says P1], but he can’t do that anymore”.*

This tendency was also marked in interview 2, where the informal caregiver participated in the interview alone, without the PwD.

*IC2: “If he was here, he would ask what was going on and sit restlessly during the whole conversation and I would calm him down when you leave. Yes, then I thought I would rather be here myself”.*

#### 3.2.2. Community Support

During the data analysis, a new theme emerged, ‘community support’, which covers a lack of awareness and understanding of PwD’s situation in public society. This is expressed in interviews 1 and 2, where both informal caregivers and P1 described situations where they have felt a lack of understanding of their situation, despite wearing a key chain that symbolizes either an invisible impairment or dementia.

*P1: “Even though I had that string on. As you get for those with Alzheimer’s”.*

*IC1: “He has it around his neck like that, but it doesn’t help when people don’t know it”.*

Similar challenges were expressed, as people do not recognize the dementia symbol, showcasing a hand.

*IC2: “It’s because his filter disappears. After all, he can also say to people, ‘Oh, you’re fat’. He was very police-like at first, not in front of people, but when we were standing at the pedestrian crossing, he said, ‘No, he must not cross, and he must not cycle there’. It was more to me that he said it, but I can’t run the risk that he will suddenly start saying it to people. He has the hand symbol of dementia on him, but no one knows that”.*

It was expressed in the interviews that it is easier for PwD and their informal caregiver to navigate in society, when people are aware of their situation and the condition.

*EM: “How do you feel about people knowing you have dementia, is it nice?”*

*P1: “Yes, it’s quite nice, I don’t take it too seriously. Then people know it”.*

*IC1: “I’d actually rather they know that than they don’t know that. Instead of them walking around and thinking, shut up, he’s a strange man”.*

*P1: “Yes, instead of what kind of person is he, messing around and standing there”.*

In one of the interviews, it was highlighted that because the dementia symbol is not recognizable in society, it was replaced by a sunflower string, which in Denmark indicates an invisible disability.

*IC2: “So last year, he got that sunflower string. He was also wearing it when we came over to the Memory Clinic. There he [the doctor] also said that it was good that he was wearing it. Yes, I then said, because no one knows the other. No, he knew that well”.*

This challenge emphasizes that, due to society’s ignorance, informal caregivers take on the role of spokespersons for PwD.

*EM: “Do you feel that people pay attention?”*

*IC2: “Both yes and no. But there was also someone who freaked out completely. We stood in line, so I told P2 that he could just go down at the other end and start packing. I don’t know what P2 said, but it wasn’t anything like that serious, but he completely freaked out. He asked if he was stupid. I also said, the man is demented, and he actually runs around with a sunflower string”.*

### 3.3. Digital Health Literacy

The analysis of the coding revealed that the codes related to the subcategories of motivation and competencies were often intertwined in the results. This overlap made it challenging to separate the two categories. Therefore, we have combined them and present them together under the term role of technology.

#### Role of Technology

Several of the participants used technology to a limited extent. Due to a lack of competence and interest in technology among the participants, several had a negative attitude towards it, and had largely written it off.

*IC1: “I have never been good at computers. I bought a new one as I had to use it and we have never been good friends. Never ever”.*

*P3: “No, I don’t have a computer here. It is with my son. I can get it here, but I have nothing to use it for”.*

*P4: “Computers are a completely closed country for me”.*

*P6: “No, I haven’t. Because I was a hairdresser, so I didn’t work with computers. It is also after my time”.*

One participant, however, showed an interest in using technology and said that he had previously used a computer in relation to his previous work. However, despite the participant’s interest in technology, specifically computers, it is highlighted several times that the participant’s primary contact with doctors and informal caregivers is not through technology.

*P5: “Yes but, sometimes via friends and yes… acquaintances and colleagues for example”.*

*EM: “Is it like talking to them, through technology?”*

*P5: “Yes”.*

*EM: “You’re borrowing a phone here, aren’t you?”*

*P5: “Hmm no, I talk to them purely telegraphically”.*

Despite the general negative attitude towards technology, all participants expressed a more positive attitude towards telephones. The participants predominantly use the telephone to call informal caregivers; here, the landline is preferred by several participants, as this is a part of a familiar routine for them.

*IC2: “No, not actually. After all, I still have my landline and it’s partly because of him that I have it too. When I’m out or something, I’ve called to say I’m on my way home. I have used the landline there. Well, he can easily figure out how to take it. He doesn’t have to do anything other than pick up the phone, he is also used to that telephone from his time”.*

Several participants also had difficulties with learning new things and routines.

*IC1: “He cannot learn new things. He can do it a little bit, the taxi thing. But in the beginning, it was very difficult. It really needs to be hammered in 1000 times”.*

Although we saw a connection between familiar routines and competencies, one of the participants also mentioned that the use of technology has become more difficult despite previously possessing competencies, which is why it requires considerable support and assistance from informal caregivers.

*P1: “He [friend] is good at electronics and such. The rest of us are no longer like that”.*

Several factors influence the use of technology, including cognitive skills.

*EM: “So it’s more small things?”*

*P1: “Yes, I can’t figure that out, that’s right”.*

*IC1: “That’s also why it’s more difficult with the phone and the iPad and things like that”.*

*EM: “So the thing about sitting and pressing small things (e.g., buttons), how is it?”*

*IC1: “Yes, phew..”.*

*P1: “Yes, it’s not so good. It was good before, but it is not anymore”.*

Technology is mainly used by informal caregivers. This is especially seen in the first interview. Here, the informal caregiver uses technology, GPS watch and find my iPhone, to keep track of P1 and feel secure in where P1 is. This highly benefits the informal caregiver by giving a sense of safety.

*IC1: “But the phone, you can see the movement more. Where the watch is, he’s standing there, and then boom, he’s over there. You can follow him completely on the phone”.*

*EM: “Okay, so you use a combination of both?”*

*IC1: “No, I use it. It is me. That watch, 1 can’t have contact with at all when he’s wearing it”.*

*EM: “P1, do you feel more secure?”*

*P1: “No, it makes my wife feel safe”.*

## 4. Discussion

### 4.1. Principal Findings

In relation to self-management, the participants had varying degrees of understanding and insight into their condition, where actual abilities and self-perception did not always match. Similarly, their social life is greatly influenced by the participants’ behavior and condition. This is expressed by several of the participants in the form of a lack of concentration, difficulty in communicating, and a feeling of quickly being overwhelmed by their surroundings.

In relation to social support, findings show that a good network has a significant impact on the participants’ opportunities for activities, facilitation of social life, and well-being. Furthermore, it was found that the informal caregivers experienced a significant burden in facilitating care and support, thus they need support and relief to maintain resources and mental energy. Additionally, a new subcategory was identified as ‘community support’, as this study has highlighted a lack of awareness and understanding of dementia in society. This makes navigation difficult for PwD and their informal caregivers. This indicates that society is not fully equipped with the necessary tools to include PwD and their informal caregivers appropriately.

The key findings within digital health literacy are that the majority of the participants have a very limited use of technology in everyday life, due to a lack of skills and aversion to technology. The participants’ use of technology was mainly seen only in the context of support from relatives or in the context of communication with relatives. Furthermore, it was found that informal caregivers predominantly used technology as a means of facilitating support.

### 4.2. Empathic Approach 

In navigating the varying degrees of dementia and the presence of informal caregivers during interviews, the use of an empathic approach proved useful. It enabled us to tailor our interactions to the unique circumstances of each participant, fostering a deeper understanding of their journeys. By embracing empathy in our research methodology, we transcended the traditional boundaries between researchers and participants, enriching our insights into the lived experiences of those affected by dementia.

Research shows that PwD require researchers in the field to be creative in their methods, reflexive in their approaches, and person-centered in their goals. Such adaptations can fundamentally change how the social practice of research is shaped and are therefore important considerations when choosing the qualitative research method [13]. Semi-structured interviews enable building a trusting relationship between the researcher and participants, which is crucial when the parties involved are affected by dementia and therefore face emotional, cognitive, and communication challenges [13]. The method is also flexible and allows adapting research questions and approaches along the way, based on new insights [21].

### 4.3. Self-Management

The participants expressed varying degrees of self-management. In particular, the participants living in nursing homes were found to have an overall lower level of self-management skills, but despite this, they still at times expressed an understanding of their health condition and a need for, e.g., doing physical activity. The low level of self-management could be attributed to the fact that these participants were all in a more advanced stage of their condition and living in nursing homes. The need to live in a nursing home partly contradicts the definition of self-management, as this is the ability to manage symptoms, treatments, and lifestyle changes needed to adapt and live with one’s condition [22].

Across all the participants, it was challenging to assess self-management, which can be partly explained by the fact that the concept does not encompass the complexity of the dementia condition. This supports a previous study by Dixon, Piper, and Lazar [23] which shows that self-management in previous literature has largely focused on neurotypical populations, leaving questions about how self-management is expressed in PwD. The involvement of informal caregivers supports self-management in PwD, as the interaction between these two parties can facilitate PwD to manage their condition. This is also consistent with the re-definition of self-management from ‘The Dementia Engagement and Empowerment Project Network’ (DEEP) [24], whereby the concept embodies a “person-centered approach where the individual is empowered and has ownership over the management of their condition and life, and a focus on the role of caregivers to support the person’s journey towards living well in the presence or absence of symptoms”.

Several of the participants needed daily physical activity, as physical activity positively affected their well-being and influenced behavioral changes such as restlessness positively. Martin et al. [25] describes that research has identified new aspects of self-management, where maintaining an active lifestyle plays a role. Despite the positive effects expressed by participants, a study by Forbes et al. [26] found that there is insufficient evidence for the effectiveness of physical activity in managing and improving cognitive function, behavior, and depression in PwD. Despite the lack of evidence between physical activity and improvement in dementia, a study by Hyde, Maher, and Elavsky [27] supports that older adults (65+ years) are motivated to stay active with a focus on maintaining their physical and mental health and supporting independence and social benefits.

### 4.4. Social Support

Support from formal and informal caregivers has a positive impact on the participants’ use of technology, their social life, and self-management. Therefore, it seems that support from the participants’ network is crucial to promote well-being. This is in line with Dixon, Piper, and Lazar [23] who demonstrate that informal caregivers and healthcare professionals need to support PwD on their journey towards living well with and without symptoms.

Support from informal caregivers varied among participants living at home with informal caregivers and participants living in nursing homes. Informal caregivers were more active in facilitating practical and social support for participants living at home compared to participants living in nursing homes, where informal caregivers acted more as a social contact. This is consistent with a previous study from 2015 by Crawford et al. [28] who found that there is a change in the role of informal caregivers when care tasks are transferred from the informal caregivers to formal caregivers, and the informal caregivers become visitors to nursing homes.

The same was seen with external support, where there was a lower need for external support among participants living in nursing homes compared to participants living at home with relatives. Here, it was mainly the informal caregivers who expressed a need for help in facilitating social and practical support. The informal caregivers described it as mentally and physically exhausting and resource intensive. This is consistent with a study by Duggleby, Schroeder, and Nekolaichuk [29] demonstrating that informal caregivers of PwD have higher levels of depression, compared to informal caregivers of individuals with intact cognitive function. The study shows that informal caregivers who do not receive adequate support during caregiving face poorer health. The additional support for informal caregivers also influenced feeling less socially disconnected, as it resulted in a greater social surplus to facilitate support for PwD. This is further supported by Drentea et al. [30] who demonstrate a link between a social support network and quality of life.

The informal caregivers also reported that behavioral changes in PwD limited the opportunities to participate in social activities, including going on trips and sitting in the park. This was expressed both in terms of a lack of desire among PwD to socialize, as well as a concern among informal caregivers for how PwD would react in social settings. This is consistent with a study by Singleton [31], which shows that dementia causes changes in behavior that can result in increased social isolation. The study also describes that caregivers need support to facilitate social integration.

In addition, we found that informal caregivers and PwD often experience a lack of understanding when interacting with other people and society, despite efforts to inform about the dementia condition in the form of symbols and support. This suggests that there is a lack of knowledge in society about the symbols of dementia and how to manage interactions with PwD. This creates a barrier for inclusion and can lead to further isolation for PwD and their informal caregivers. This is consistent with Singleton’s study [31], which describes that there are negative consequences when dementia is not a recognized factor in the behavioral changes that cause challenges in social contexts.

### 4.5. Role of Technology

For most of the participants, technology plays a minor role in their everyday lives. None of the participants use a computer and the use of technology was mainly seen as a means of contact and communication with informal caregivers. Here, it was expressed that it was often the informal caregivers calling them, which again shows the low interaction with technology among PwD. In addition, only one participant had a smartphone, while the rest had a mobile phone or a landline phone. A study by Statistics Denmark from 2019 shows that the older generation, in the age group 75–89 years old, use the internet the least; in addition, 17% of this group are not users of the internet [32]. In the same study, 33% in the same age group, equivalent to every third person, believe to a lesser extent or not at all that they have enough knowledge about IT security, which can lead to insecurities in the use of technology [32].

Several participants used their age as a reason for not using technology, which seemed to lead to less motivation to use technology as they did not feel comfortable navigating it. This is supported by a study by Terp, Kayser, and Lindhardt [33] who, in a study of older patients’ technology readiness, identified that the participants’ perception of their competencies, especially related to their age, created a lack of confidence and a barrier to their motivation to use technology. However, it can be argued that it is not only age that influences the use of technology, as a study by Dahlke and Ory [34] shows that socioeconomic status also has an impact on the extent and type of technology that is an integral part of everyday life.

A greater extent in the use of technology was seen by both the formal and informal caregivers, but it was expressed several times that the technology was used to assist informal caregivers in connection with PwD. One of the informal caregivers stated that the technology used is designed to assist them in taking care of and having an overview of the PwD. It can therefore be challenging to assess what needs one gains insight into when it comes to the use of technology, especially since much of the technology developed to support dementia is developed to assist informal caregivers. This is further supported by a 2021 study by Dixon, Piper, and Lazar [23] that technology is mainly developed to support informal caregivers and PwD in the home, especially to help with the task of navigation to prevent PwD from wandering. A study by Robinson et al. [35] on the development of assistive technologies describes the importance of involving PwD in the design of technologies, as they, despite challenges with cognitive skills, want to maintain their autonomy. Technological solutions must be designed for PwD to use, for example, to find their way home and be able to adjust as their condition deteriorates. Furthermore, Robinson et al. [35] highlights that stigmatization is reduced and independence is promoted when technologies are less visible to the outside world.

## 5. Limitations and Strengths

A limitation of this study is the low number of only seven PwD participating in the interviews, which may challenge the generalizability of the findings. Further studies are needed with the inclusion of more participants, preferably with a wider age range, variations in living status, and prior and current experience with technology. Access to such data would enable triangulation of these socio-demographic characteristics with the participants’ responses. A larger study extending into PwD with mild to moderate cognitive impairment would also make it possible to use the quantitative READHY instrument to characterize the participants [33], which is not possible with PwD with moderate to severe cognitive impairment. This study focused on PwD with moderate to severe cognitive impairment, and we found it difficult to recruit from this group. Thus, we decided to report on the seven PwD together with two informal caregivers living with those two who did not live in a nursing home, as we find that our study has sufficient information power [36]. Our argument is that the selected population has resulted in lower variation, as the seven PwD share common characteristics such as having moderate to severe cognitive impairment, being older, and living with support in their everyday lives. Additionally, several similarities in expressed needs and challenges were found, e.g., the need for physical activity, little use of technology, and social disability. Thus, the relatively homogenous sample should have enough information power to propose a new way to approach PwD with moderate to severe cognitive impairment and demonstrate how the READHY framework can be used to investigate their technology readiness and needs. This is also supported by the similarity between our findings and previous reports. 

A strength of this study is the use of an empathic approach, which made it possible to connect with the participants and gain a better understanding of their perspectives (15) and a deeper insight into their experiences and descriptions of needs, challenges, and feelings. This contributed to a person-centered focus in the qualitative analysis of the data. Additionally, the utilization of the READHY framework facilitated a comprehensive analysis of the qualitative data, providing rich insights into the experiences and perspectives of PwD.

## 6. Conclusions

Although recruiting PwD for this study presented challenges, our research methodology, grounded in empathy, allowed us to surpass conventional researcher–participant boundaries in both recruitment and conducting the interviews. This approach significantly deepened our understanding of the lived experiences of those affected by moderate to severe dementia, providing richer and more meaningful insights. PwD have a complicated relationship with digital technologies, and using these services can be confusing and overwhelming without the right support. Consistent support from informal caregivers can have a positive impact on the lives of PwD, especially since several participants feel insecure and distrust both formal and informal caregivers at times in their daily lives. Support from informal caregivers can positively impact the PwD’s opportunities for social activities and well-being, as the results show that it is primarily informal caregivers who facilitate practical and social support for PwD living at home. However, informal caregivers experience a high burden in facilitating support to PwD. Therefore, it is important to address that informal caregivers need support to maintain their mental energy and resources to avoid negative effects on their well-being and social lives. Digital technologies can be integrated to support independence among PwD and lessen the burden on informal caregivers. The current use of technology is limited for PwD, which calls for new ways to engage and collaborate with PwD and their informal caregivers. An empathic approach can facilitate a deeper insight into individual needs and competencies, which can be used to design more accessible, inclusive, and non-intrusive technologies, building on routines familiar to the PwD.

## Figures and Tables

**Table 1:** Participants.

Participants	Gender	Age	Recruitment Method
Participant 1 (P1)	Male	68	Colleague referral
Informal caregiver (IC1)	Female	N.R.	Colleague referral
Participant 2 (P2)	Male	74	Community event
Informal caregiver (IC2)	Female	N.R.	Community event
Participant 3 (P3)	Female	78	Personal contact to nursing home
Participant 4 (P4)	Female	97	Personal contact to nursing home
Participant 5 (P5)	Male	58	Personal contact to nursing home
Participant 6 (P6)	Female	102	Personal contact to nursing home
Participant 7 (P7)	Female	92	Personal contact to nursing home

N.R.: Not registered.

**Table 2:** Code tree.

Main Category	Subcategories	Code
Self-management	Impact of condition - heiQ4: Constructive attitudes and approaches - heiQ8: Emotional distress	- Affected by condition including well-being - Emotional reactions related to the dementia condition
	Capabilities and Resources - heiQ3: Self-Monitoring and insight - heiQ5: Skills and technique acquisition	- Attitude and mental coping with the condition - Insight and acknowledgment of limitations, as well as the ability to adhere to them - Lack of understanding of abilities, skills, and health - Treatment, aids, and techniques for managing condition-related symptoms and health problems
Social Support	Internal Support - HLQ4: Social support for health	- Limitations or challenges regarding support from family and friends - Emotional support or feeling understood - Practical support from family and friends - Social life
	External Support - HLQ1: Feeling understood and supported by healthcare providers	- Feeling understood, supported, and considered by healthcare professionals regarding help, guidance, and counseling - Challenges and limitations regarding trust and confidence in healthcare professionals and the system - Relevant services and respite options
	Community Support	- Community support
Digital Health Literacy	Competencies - eHLQ3: Ability to actively engage with digital services - eHLQ6: Access to digital services that work - eHLQ7: Digital services that suit individual needs	- Competencies
		- Use and context of existing technology
		- Limitations or challenges related to technology
	Motivation - eHLQ5: Motivated to engage with digital services	- Attitude and motivation towards the use of technology

## Data Availability

The datasets used and/or analyzed during the current study are available from the corresponding author upon reasonable request.

## Supplementary Materials

The following supporting information can be downloaded at: https://www.mdpi.com/article/10.3390/ijerph21081023/s1, Table S1—Interview guide for the semi-structured interviews.
