# Supplementary material for: Using an Empathetic Approach to Explore Technology Readiness and Needs for Digital Services to Assist People with Dementia
Source: Int J Environ Res Public Health. 2024 Aug 2;21(8):1023. doi: 10.3390/ijerph21081023 (PMC11354846; doi:10.3390/ijerph21081023)
Supplement: Supplementary file 1 [file ijerph-21-01023-s001.zip › ijerph-3067502-supplementary.pdf]

Table S1 - Interview guide for the semi-structured interviews

<table>
<tr><td colspan="3">Interview Guide</td></tr>
<tr><td colspan="3">Introduction
We would like to start by saying thank you for being willing to be a part of this conversation.

Our names are XXX and XXX. We study Health and Informatics at the University of Copenhagen, where we are working on a major final project. Our project focuses on people with dementia, where we will explore challenges related to social activities and general well-being, as well as examine the use of technology. Therefore, we would like to talk to you about your experiences and thoughts.

Information from the conversation will be anonymized so that you cannot be recognized from the project or any subsequent article. If there is anything you are unsure about or want to know more about during the interview, please feel free to ask. The interview will last approximately 30-60 minutes. Is that okay with you? If it is, we would like to record the conversation so that we can work on it later. We will delete the recording once the project is completed.

If you are ready, we will start the conversation. I'll press start so we can begin. To start, we can tell you a bit about ourselves.</td></tr>
<tr><td>Theme</td><td>Subtheme</td><td>Central topics (Check-up)</td></tr>
<tr><td>Introductory questions</td><td>Who is the interviewee?</td><td>• Age
• Gender
• Marital status
• Family/children
• Living situation</td></tr>
<tr><td>The individual's own mastery and resourcefulness in managing their own health</td><td>What understanding does the interviewee have of their own mastery of health and how is it managed?</td><td>• Symptoms
• Challenges
• Skills/competencies
• Treatment/medication</td></tr>
<tr><td>The feeling of support from networks such as family members and healthcare professionals</td><td>What experience does the interviewee have of their network in everyday life, do they feel they receive the support they need?</td><td>• Family/friends
• Healthcare professionals
• Understanding
• Forms of contact</td></tr>
<tr><td>Digital health literacy</td><td>What relationship/skills does the interviewee have with technology, is there an interest and value in the use of new/existing technology in everyday life?</td><td>• Motivation/interest
• Extent/use of technology Utility value
• Support</td></tr>
<tr><td>The individual's own mastery and resourcefulness in managing their own health</td><td>How has the interviewee's perception of their own well-being and health changed over the past years, and how do they assess their current and future well-being and health?</td><td>• Assessment of well-being
• Assessment of health</td></tr>
</table>
